# Trends in Vaccine Completeness in Children Aged 0–23 Months in Cape Town, South Africa

**DOI:** 10.3390/vaccines11121782

**Published:** 2023-11-29

**Authors:** Duduzile Ndwandwe, Musawenkosi Ndlovu, Asanda Mayeye, Nomahlubi Luphondo, Ndivhuwo Muvhulawa, Yonela Ntamo, Phiwayinkosi V. Dludla, Charles S. Wiysonge

**Affiliations:** 1 Cochrane South Africa, South African Medical Research Council, Tygerberg, Cape Town 7505, South Africa; musawenkosi.ndlovu@mrc.ac.za (M.N.); asanda.mayeye@mrc.ac.za (A.M.); nomahlubi.luphondo@mrc.ac.za (N.L.); mn.muvhulawa@gmail.com (N.M.); yonela.ntamo@mrc.ac.za (Y.N.); pdludla@mrc.ac.za (P.V.D.); 2 Department of Biochemistry, North-West University, Mafikeng Campus, Mmabatho 2735, South Africa; 3 Department of Biochemistry and Microbiology, University of Zululand, KwaDlangezwa, Empangeni 3886, South Africa; 4 Vaccine Preventable Diseases Programme, Universal Health Coverage/Communicable and Non-Communicable Diseases Cluster, World Health Organization Regional Office for Africa, Brazzaville P.O. Box 06, Congo; sheyc@who.int

**Keywords:** missed opportunities for vaccination, vaccination, immunisation, children, primary health care, quality improvement, South Africa

## Abstract

Background: We have previously determined that the occurrence of missed vaccination opportunities in children in Cape Town, South Africa, is shaped by both individual and contextual factors. These factors present valuable openings for enhancing quality and implementing broader strategies to enhance the delivery of routine Immunisation services. Methods: Here, we are further reporting regional-level data on the coverage and factors influencing vaccination completion within a similar study population, based on extensive data analysis from the 2016 South African Demographic and Health Survey. Results and discussion: The study reveals commendable vaccination coverage for most vaccines within recommended schedules, with high rates of initial vaccinations at birth and during the primary vaccination schedule. However, there are notable areas for improvement, particularly in ensuring complete coverage for the second measles vaccine and the 18-month vaccine. Socio-demographic factors also play a role, with maternal education and caregiver awareness campaigns showing the potential to positively influence vaccination completeness. This study emphasises the importance of timely vaccinations during the early months of life and underscores the need for interventions to maintain coverage as children age. Specific sub-districts, such as Tygerberg, may require targeted efforts to enhance vaccination completeness. Additionally, assessing caregiver knowledge about child vaccination is deemed vital, as it can impact vaccination decisions and adherence. Conclusions: The findings provide valuable insights for public health interventions in Cape Town, aimed at reducing the burden of vaccine-preventable diseases and ensuring the health of the region’s youngest population.

## 1. Introduction

Childhood vaccination is one of the most critical tools at our disposal during the ongoing global effort to combat infectious diseases and safeguard public health [1,2]. Vaccination protects individual children from life-threatening illnesses and contributes significantly to communities’ collective immunity, preventing the spread of diseases [3]. Childhood immunisation is universally recognised as one of the most cost-effective public health interventions, significantly reducing morbidity and mortality rates among children [4]. Vaccines bolster the immune system of infants and young children against various preventable diseases, such as measles, polio, diphtheria, pertussis, and hepatitis B [5]. The importance of childhood immunisation extends beyond individual protection; it forms the cornerstone of herd immunity, where a sufficiently high vaccination rate within a community prevents the outbreak and spread of diseases [6]. Vaccine integrity encompasses the quality and effectiveness of vaccines throughout their lifecycle, ensuring stability from production to administration [7]. Monitoring vaccine integrity trends involves analysing changes and improvements in vaccine technology over time [8]. Herd immunity, on the other hand, is achieved when a substantial portion of a population becomes immune to a specific disease, either through vaccination or previous infection, thereby slowing or halting the disease’s spread and providing indirect protection to those who are not immune [9]. These concepts are particularly relevant to determine in densely populated urban areas like Cape Town, where infectious diseases can quickly gain a foothold and escalate into epidemics if vaccination rates fall below the threshold required for herd immunity [10,11].

Cape Town, located in the Western Cape province of South Africa, grapples with socioeconomic disparities mirrored in its healthcare landscape [12]. The socio-economic diversity, high population density, and significant healthcare disparities create a complex backdrop for the study of childhood immunisation trends. For example, it has already been acknowledged that concerns implementing “side effects”, “doubts about its effectiveness”, and “being opposed to vaccines in general” are the most common reasons given by South Africans who worry about becoming vaccinated [13]. South Africa has a history of successful vaccination campaigns and has made significant strides in recent decades, particularly in reducing the incidence of vaccine-preventable diseases [14]. This achievement is largely contributed to by introducing programs like the Expanded Program on Immunisation (EPI), which focuses on providing vaccines to children during their first year of life [15,16]. Nevertheless, South Africa, like many African countries is still faced with persistent challenges, including but not limited to vaccine hesitancy [17,18,19,20], unequal access to healthcare [21], and logistical difficulties in reaching remote and underserved communities [22]. These challenges have implications for vaccine completeness in the country and specific regions like Cape Town.

In Cape Town, as in many urban areas worldwide, a stark contrast exists between affluent neighbourhoods with well-established healthcare infrastructure and impoverished townships struggling with limited access to quality healthcare services. Additionally, cultural beliefs and education levels within communities also impact vaccine completeness. Just like in the broader South African context, in Cape Town, there are diverse cultural backgrounds and beliefs that potentially influence vaccination decisions. For example, in the context of coronavirus disease (COVID-19), previous research undertaken among the South African adult population has indeed identified race, interactive critical vaccine literacy, and trust in the government’s ability as key factors influencing the uptake of vaccination [23,24]. Moreover, misconceptions, such as unfounded concerns about safety or efficacy, as well as general myths, may also drive vaccine hesitancy [4,25]. The COVID-19 pandemic also significantly affected the healthcare systems, including childhood immunisation programs worldwide [23,24]. In Cape Town, routine immunisation services were disrupted. 

Our previous research has already reported on missed opportunities for vaccination among children in Cape Town [4,25]. The results from this study are mainly focused on determining various factors such as healthcare system failures, lack of awareness, or vaccine hesitancy, while also identifying gaps in the delivery of vaccines, aiming to improve vaccination rates and public health outcomes in Cape Town. However, there is also an evidence gap regarding vaccine completeness within this research niche. This includes determining the extent to which an individual or a population has received the full recommended course of vaccinations for a particular disease. And further assessing whether children have received all the required doses at the appropriate intervals to establish strong immunity. Thus, this study builds on from our previous research [4,25] but focuses on vaccine completeness in children from birth to 23 months in Cape Town, including assessing the evolving trends that impact their health and well-being [4,5,25]. 

This research will shed light on the progress made and the challenges that still lie ahead in ensuring comprehensive vaccine coverage for children in this dynamic urban landscape. More so, Cape Town’s diverse population offers a unique opportunity to explore how cultural and educational factors intersect with vaccine completeness trends [26,27,28]. Long wait times, inadequate staffing, and vaccine supply chain management challenges can hinder vaccine delivery [28,29,30]. As such, examining the role of healthcare access and infrastructure is essential for devising strategies to overcome these barriers and improve vaccination rates.

## 2. Materials and Methods

### 2.1. Study Design and Setting

A thorough investigation was conducted to examine the patterns of childhood Immunisation and the factors that lead to incomplete vaccination. This was accomplished through a cross-sectional, multi-level modelling technique, which was based on the 2016 South African Demographic and Health Survey (SADHS) [31]. To accurately address the complex nature of the data and the possible grouping of personal characteristics within healthcare facilities, a multi-level analytical approach was deemed necessary. As described by Nnaji et al. [25], the data used in this study were collected from Primary Health Care Facilities (PHCF) situated in five health sub-districts of the Cape Town Metropole between 3 August and 15 October 2021. These subdistricts included Khayelitsha, Michells Plain, Southern, Tygerberg, and Western. Extending from previous studies [25,32], the selection and motivation for choosing these subdistricts is because they remain an important part of the Cape Town Metropolitan Health District, serving an estimated population of 4.1 million persons. The significance of evaluating vaccine completeness for children with an approximate age of 2 years is already established elsewhere [33,34]. The present study adhered to the current South African childhood immunisation schedule as recommended by the World Health Organization (WHO), as previously reported [4,25]. Importantly, data from the 2016 South African Demographic and Health Survey remain crucial for providing a comprehensive understanding of the health status of the country [31]. Child health, encompassing vaccine completeness, remains integral to assist the Department of Health to plan and prioritize health programmes and service delivery in South Africa. 

### 2.2. Study Population and Sample Size

The study population has already been described [4]. Briefly, this study included caregivers of children from birth up to 23 months attending PHC facilities across the five districts on the Cape Town Metro, for various health services on the day of assessment. The eligibility criteria included the child’s presence at the PHC facility brought by the caregiver aged ≥18 years. A total of 674 caregivers participated in the study. Sample size estimation was based on a previous study already indicating that a minimum size of 630 participants is appropriate to give an alpha level of 0.05 (95% confidence interval), a margin of error of 5%, non-response rate of 20%, and a design effect of 1.5 [25,35].

### 2.3. Data Collection

Data were collected and managed using Research Electronic Data Capture (REDCap) through exit interviews using an electronic structured and interviewer-administered questionnaire, as previously described [25]. Briefly, the questionnaire had six sections that helped assess vaccine completeness: Section A related to data on the child; Section B on the parent/caregiver; Section C on the use of the Road to Health Booklet (RtHB) for checking vaccination status and information on vaccine administered; Section D on current visit; Section E on quality of the vaccination service; and Section F on reasons for vaccination. Moreover, trained research assistants who were fluent in English, Afrikaans, and IsiXhosa were responsible for data collection and conducting exit interviews. Importantly, data collectors were stationed strategically at the main exits of each health facility, to easily approach caregivers accompanied by children as they were exiting the facility.

### 2.4. Data Analysis

The collected data were cleaned and coded, using an Excel spreadsheet from Microsoft and STATA statistical software version 15.0. A descriptive analysis was performed to generate the frequency and percentages of the independent variables by the outcome variables (full and incomplete vaccination). Logistic regression analysis (bivariable and multivariable) was performed to determine the presence of an association between the dependent and independent variables. The bivariate analysis was used to examine the crude association between each independent variable and childhood Immunisation (full and incomplete vaccination), while the multivariate analysis was used to assess the adjusted association between the independent variables and childhood Immunisation. In both bivariable and multivariable logistic regression analysis, measurements of association were presented as odds ratio (OR) with their corresponding 95% confidence intervals (CI), and variables with a *p*-value of less than 0.05 were considered significant.

### 2.5. Study Variables

The two dependent variables were full vaccination and incomplete vaccination. Fully vaccination referred to any child aged 0–23 months old who received all vaccine doses recommended by the WHO, while incomplete vaccination referred to any child aged 0–23 months old who missed at least one of any doses of the routine vaccine per the WHO recommendations. Independent variables, selected based on our previous study [25], included child-related factors such as age, sex, birth order, and birth weight, as well as caregiver-related factors like marital status, employment status, maternal age, relationship with the child, level of education, receiving vaccination messages in the last 3 months, mode of transport to the health facility, attendance of antenatal care. Health facility-related factors were facility type, ownership, number of health workers, vaccine stock-out in the past three months, and vaccine cold-chain challenges in the past 3 months. Briefly, in terms of “total vaccination” and “incomplete vaccination”, a child was considered fully vaccinated if the child completed the vaccination schedule by receiving all WHO-recommended vaccine doses, while incomplete vaccination refers to any child who has missed any of the vaccines as recommended by the WHO. In this study, children who visited the health facilities and received all vaccine doses were considered fully vaccinated and those who missed at least one vaccine dose were referred to as incompletely vaccinated. 

## 3. Results

**Figure 1:**
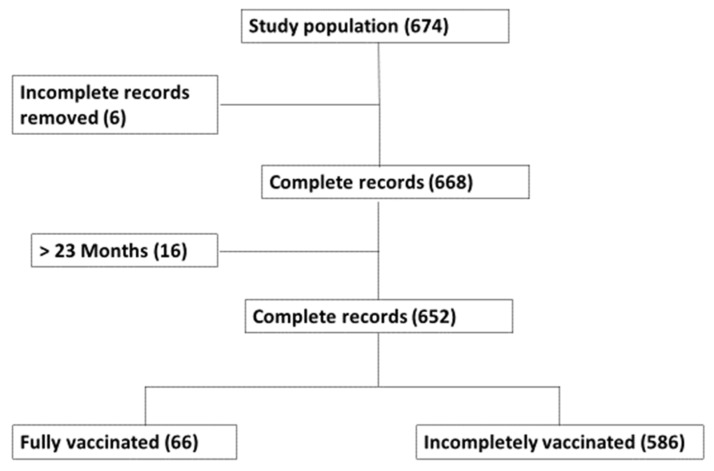
Flowchart for the identification of missed opportunity vaccination among children from birth to 23 months in Cape Town.

The present study sampled 674 participants across five sub-districts within 11 PHC facilities in Cape Town metropolises. The study population only focused on children from birth to 23 months. Children above 23 months old and incomplete records were excluded from the analysis. A total of 652 records were found eligible for vaccine completeness analysis and, of these, only 66 children were fully vaccinated while 586 were incompletely vaccinated (Figure 1).

**Figure 2:**
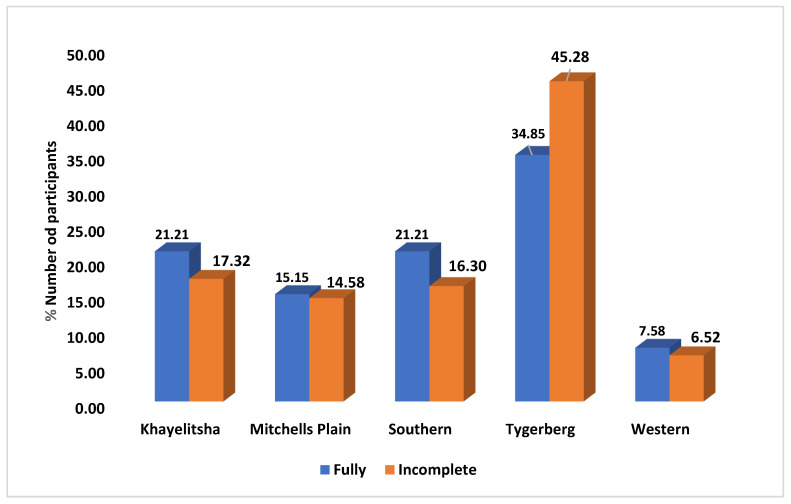
Distribution of all fully vaccinated and incompletely vaccinated participants across the five sub-districts within 11 PHC facilities in Cape Town metropolises. The subdistricts accessed included Khayelitsha, Michells Plain, Southern, Tygerberg, and Western.

After collecting the complete records, we further looked at the vaccine “completeness” among the different sub districts. The Tygerberg sub-district had the highest number of records, with 287 (44%) participants. Of these participants, 264 (45.28%) were incompletely vaccinated, while only 23 (34.85%) were fully vaccinated. Meanwhile, the Khayelitsha sub-district recorded the second highest number of participants, 115 (18%), of which 101 (17.32%) were incompletely vaccinated and 14 (21.21%) were fully vaccinated. These findings indicate that Tygerberg had the largest number of participants in the study, with many of them being incompletely vaccinated. Conversely, while Khayelitsha had the second highest number of participants, a higher percentage of them were fully vaccinated compared to Tygerberg (Figure 2).

### 3.1. Dose-Specific Vaccination Prevalence 

From the complete records (652), we further assessed dose-specific vaccination prevalence. Table 1 examines two vaccines administered at birth BCG and OPV. The data indicate that the vaccination rate for BCG is 98%, with only 1.53% of children not vaccinated and five children whose vaccination status is uncertain. Similarly, OPV (0) had a vaccination rate of 96.63%, with 2.3% not vaccinated and seven children with an uncertain status. As the table progresses to the 6-week mark, it evaluates multiple vaccines, including OPV (1), RV (Rotavirus Vaccine), DTaP-IPV-Hib-HepB (Hexavalent Vaccine), and PCV (Pneumococcal Conjugate Vaccine). These vaccines generally have high vaccination rates, with only a few percentage points of children not vaccinated and a small number with an uncertain status. Table 1 shows that data follow a similar pattern in tracking vaccination rates and missed doses at 10 weeks, 14 weeks, 6 months, 9 months, 12 months, and 18 months. Some vaccines, such as measles (1) at 6 months and measles (2) at 12 months, have lower vaccination rates, with a higher percentage of children not vaccinated. Overall, Table 1 also provides a comprehensive snapshot of the vaccination landscape for children in Cape Town. It shows the successes in achieving high vaccination rates for most doses, as well as the challenges in ensuring complete coverage, particularly for certain vaccines at specific age milestones. This information is valuable for public health officials and policymakers, as it identifies areas where targeted interventions may be necessary to improve vaccination rates and secure the health of the community’s youngest members.

### 3.2. Socio-Demographic Characteristics of the Mother/Caregiver and the Child

#### 3.2.1. Influence of Basic Demographic Characteristics on the Vaccination Status

Table 2 offers a detailed examination of the demographic characteristics of both the mother/caregiver and child, highlighting their correlation with incomplete childhood vaccination among children from birth to 23 months who visited primary healthcare facilities in Cape Town, South Africa. The study included 652 children, with males and females being almost equally represented (49% and 51%, respectively). However, when it came to vaccination status, a significant majority of incompletely vaccinated children were male (89%). Most of the children (76%) were aged 10 months or younger, and it is noteworthy that most of the fully vaccinated children were also in this age group. The study uncovered that children were born in different birth orders, with firstborns making up 36% of the sample. Furthermore, fully vaccinated children were more prevalent among firstborns (9%). While 63% of children had an average birth weight, and there were more fully vaccinated children within this group compared to other birth weights (Table 2).

#### 3.2.2. Influence of Vaccination Visit on the Vaccination Status

The primary reason for most healthcare facility visits was vaccination, making up 67% of all visits. Interestingly, incompletely vaccinated (91%) children were seen more frequently for this purpose than those who were fully vaccinated (Table 2). Almost all children, at 98%, were asked about their Right to Health Booklet (RtHB) by a healthcare worker. The majority of the visits took place in the morning, with fully vaccinated children representing 10.43% and incompletely vaccinated children making up 90%. Afternoon visits were less frequent, with fully vaccinated and incompletely vaccinated children representing 10% and 90%, respectively (Table 2).

#### 3.2.3. Influence of Caregiver Position, Education Level, and Employment Standing on the Vaccination Status

The study encompassed mothers and caregivers of diverse age groups, among whom fully vaccinated children were found to be slightly more prevalent among those aged 25–34 years (11%). Most caregivers were female (630 out of 652 children), and only a small percentage of fully vaccinated children had male caregivers (Table 2). The marital status varied, with single mothers or caregivers being more prevalent, but fully vaccinated children were found to be slightly more common among those who were married (11%). Out of 652 children, the majority (93%) had their mothers as primary caregivers, with the remaining caregivers being fathers and siblings. Of these mothers, 91% had incomplete vaccination records for the child and 9.44% had fully vaccinated children (Table 2). The education levels of the caregivers varied, with the majority having secondary education. However, fully vaccinated children had a slightly higher percentage of mothers with post-secondary education (10%). Some mothers or caregivers were employed, while others were not. Interestingly, a slightly higher percentage of fully vaccinated children had employed mothers or caregivers (13%). Almost all mothers or caregivers (99%) received antenatal care, with a slightly higher percentage of fully vaccinated children having mothers or caregivers who received antenatal care (Table 2).

#### 3.2.4. Influence of Transport during Vaccination Visit and Facility Characteristics on the Vaccination Status

The mode of transport used to travel to healthcare facilities differed, with private, public, and walking being common methods. A higher percentage of fully vaccinated children used private transport (9%). Most children received an immunisation message in the last 3 months (88% incompletely vaccinated and 12% fully vaccinated) (Table 2). The children attended different healthcare facilities, including clinics and Comprehensive Disease Control (CDC) centres. Vaccination rates were similar in both types of facilities, and the facilities were either owned by the province or the city, with similar vaccination rates between the two ownership categories. Facilities had varying numbers of health workers, with facilities that had fewer than 20 health workers having a higher percentage of fully vaccinated children (13%). Some facilities encountered vaccine stock-outs in the past 3 months (10% fully vaccinated facilities), but the majority did not face this issue. Few facilities experienced vaccine cold-chain challenges (10% fully vaccinated facilities), but the majority did not (Table 2).

### 3.3. Mother/Caregiver Related Factors

**Figure 3:**
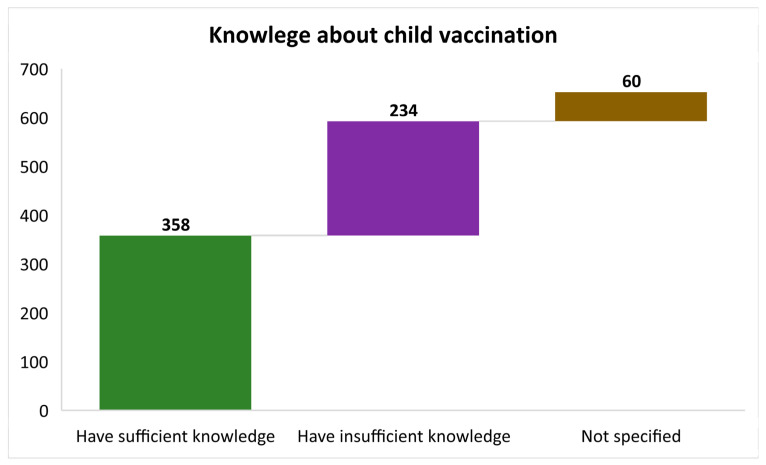
Knowledge about child vaccination among mothers or caregivers presented in bars showing those who had sufficient knowledge, insufficient knowledge, and those who did not answer indicated as “Not specified”.

Figure 3 offers a visual representation of the knowledge surrounding child vaccination among mothers or caregivers. The data are presented in bar form, with each segment representing a distinct category of knowledge. The first segment (in green colour) displays those with adequate knowledge about child vaccination, including details such as their importance, schedule, and significance. The second segment (purple colour) represents mothers or caregivers with incomplete or inadequate knowledge about child vaccination. They may have gaps in their understanding of vaccination-related topics. Lastly, the third segment (brown colour) encompasses those who did not provide a response or answer to questions regarding their knowledge of child vaccinations. By examining the distribution of mothers or caregivers across these three categories, Figure 3 provides valuable insights into the level of awareness and understanding surrounding child vaccination within the studied population, helping to identify areas where education and awareness campaigns may be needed to improve vaccination knowledge among caregivers.

### 3.4. Measurement of Factors Associated with Missed Opportunity Vaccination

Table 3 presents an analysis of factors associated with vaccine completeness among children aged 0–23 months in five districts in Cape Town, South Africa. The table includes various variables and association measurements, both bivariate (crude) odds ratios and adjusted odds ratios, along with their respective *p*-values. The table indicates that female children had slightly lower odds of vaccine completeness, but this difference was not statistically significant. Older children (11–23 months) had much lower odds of experiencing full vaccination compared to infants aged 10 months or less (Table 3). This effect remained significant (*p* < 0.001) even after adjusting for other factors. The child’s birth order did not significantly impact the likelihood of vaccine completeness. Birth weight did not show a significant association with vaccine completeness. Children who visited healthcare facilities for non-vaccination reasons had higher odds of incomplete vaccination, but this difference was not statistically significant (Table 3). The time of the healthcare facility visit (morning or afternoon) did not significantly impact on vaccine completeness.

Maternal age did not show a significant association with vaccine completeness (Table 3). Children with female caregivers had higher odds of vaccine completeness, but this difference was also insignificant. The marital status of the mother or caregiver did not significantly impact on vaccine completeness (Table 3). The caregiver’s relationship with the child did not show a significant association with vaccine completeness (Table 3). Maternal education levels did not significantly impact child vaccination. The employment status of the mother or caregiver did not significantly impact child vaccination status (Table 3). The presence or absence of maternal antenatal care, including the means of transport to reach healthcare facilities did not significantly impact child vaccination (Table 3). Children whose caregivers did not receive an Immunisation message in the last 3 months had higher odds of vaccine completeness, but this difference was not statistically significant (Table 3). The type of healthcare facility (clinic or CDC) did not show a significant association with childhood vaccination status (*p* < 0.05) (Table 3). However, facilities owned by the province or the city did not significantly impact child vaccination. The number of health workers in the facility did not show a significant association with children vaccination. Vaccine stock-out or cold-chain challenges in vaccine storage did not significantly impact children vaccination. The waiting time for Immunisation did not show a significant association with children vaccination. Therefore, the data presented in Table 3 provide an in-depth analysis of various factors associated with children vaccination among children in Cape Town, South Africa. While some variables showed trends, most of the associations were not statistically significant, suggesting that child vaccination may be influenced by complex factors not captured in this analysis. Further research and targeted interventions may be needed to address MOV effectively in this context.

## 4. Discussion

This study evaluated the complex landscape of childhood vaccination completeness in Cape Town, South Africa, based on a comprehensive analysis of our data, and from our previously published findings [4,25]. The current findings focus on the vaccination coverage rates for various vaccines, the socio-demographic factors influencing incomplete childhood vaccination [11], and the geographical distribution of vaccination records across sub-districts, including Khayelitsha, Michells Plain, Southern, Tygerberg, and Western [22]. By examining these facets, this study aims to unravel the strengths and challenges within the vaccination program, pinpointing areas where interventions are needed to enhance vaccine coverage, ensure timely Immunisation, and safeguard the health of the region’s youngest population.

Vaccination coverage rates among children aged 0–23 months in Cape Town, South Africa, for different vaccines at different ages. Crucial insights into the prevalence of vaccination, as previously reported [4,25], highlights that most children received the birth doses, with over 97% vaccinated, which is a positive sign. These initial vaccines protect newborns from dangerous diseases such as tuberculosis and polio. The vaccination coverage rates remain high for vaccines administered at 6 and 10 weeks of age, with over 92% and 98% of children receiving these vaccines, respectively (Table 2). The high vaccination coverage rates for initial vaccines (BCG and OPV) and vaccines administered at 6 and 10 weeks in the study population can be attributed to several factors. Firstly, a substantial proportion of children (76%) received vaccinations at an age of 10 months or younger, indicating early and timely Immunisation. This is a positive sign as it aligns with recommended vaccination schedules [36]. Furthermore, most mothers or caregivers (93%) had a direct relationship with the child, likely contributing to a sense of responsibility for the child’s health. Maternal education also played a role, with a significant percentage (69%) having at least a secondary education, indicating a potential correlation between education and awareness of the importance of vaccinations. Interestingly, this aligns with a systematic review and meta-analysis indicating that improving maternal education is important for increasing childhood vaccination uptake and coverage [37]. Additionally, the high rate of maternal antenatal care (98%) suggests a proactive approach to healthcare, which likely extends to Immunisation. Facility-related factors, such as the absence of vaccine stock-outs and cold-chain challenges in the past three months, further contribute to the positive vaccination coverage. 

These findings highlight the importance of early initiation, maternal education, and healthcare infrastructure in achieving high vaccination rates. This may potentially indicate that the primary vaccination schedule is generally followed properly. Our data confirm previous findings that the prevalence of vaccination is highest for the second dose of measles vaccine (9.5%) and lowest for the first dose of rotavirus vaccine (0.6%) [4,25]. In general, there are various factors that influence the vaccination status of a child, as such the likelihood of a child experiencing vaccine-preventable disease, which may correlate with caregivers having a low level of education, not receiving recent Immunisation messages, or perhaps having shared Immunisation decision making by both parents and health facility staff number [25].

We found that vaccination coverages differed based on the various schedules. For example, at 14 weeks, vaccination coverage remains relatively high, with around 96% of children receiving the vaccines. However, at 6 months, the coverage for the measles vaccine dropped slightly to 95%, indicating that there might be a need for increased efforts to ensure timely measles vaccination. Additionally, our data showed that at 9 months, the coverage for the PCV 3 vaccine was around 97%, indicative of effective vaccine administration. Further concerns or rather irregularities were evident in our data, as at 12 months, the measles dose 2 coverage dropped to 91%, indicating a potential gap in ensuring children receive their second measles vaccine. Like the second dose of measles which is administered at 12 months, the coverage for the DTaP-IPV-HepB vaccine at 18 months was 89%, indicating that there may be room for improvement in completing the vaccination series by this age. This phenomenon has been observed to illustrate that vaccine coverage is dependent of age [38]. Guyer et al. [38] assessed the expanded Immunisation programme during the first 9 months, where approximately 22,000 children were Immunised against poliomyelitis, measles, tuberculosis, smallpox, whooping cough, tetanus, and diphtheria. This showed that the rates of Immunisation coverage in the target population; 30% for DPT (one dose or more), 27% for poliomyelitis (one dose or more), 27% for BCG, 33% for measles, and 20% for smallpox. Eighty per cent of children received the correct vaccines for their age and vaccination status. Seroconversion to measles vaccine was 89% in those over 12 months of age but only 50% in those between 6 and 11 months of age in Cameroon [38]. 

Thus, our findings suggests that Cape Town has achieved commendable coverage rates for most vaccines within the recommended schedules. However, there are some areas, such as the second measles vaccine and the 18-month vaccine, where coverage can be further improved. Notably, there may be a potential gap in the vaccination schedule or a delay in administering certain vaccines, warranting further investigation into the specific causes of this deviation from the expected coverage. This pattern may signify challenges in sustaining timely vaccinations as children grow older, especially in developing countries like those within sub-Saharan Africa. As previously reviewed [39], possible factors contributing to these observations could include issues related to awareness, accessibility, or healthcare system efficiency. Addressing these discrepancies is crucial for ensuring comprehensive and timely Immunisation coverage throughout the recommended vaccination schedule. 

An overview of the socio-demographic characteristics of mothers or caregivers and children, including their association with incomplete childhood vaccination was also evaluated. The data highlight that children aged 11–15 months had a higher percentage of complete vaccination (4.3%) compared to children aged ≤ 10 months (0.42%). This indicates the importance of age as a determinant of vaccination completeness, with older children having a better chance of being fully vaccinated. Interestingly, children who were the firstborn in their families had a slightly higher rate of complete vaccination (9%) compared to those who were second (8%) or third-born (13.39%). But, surprisingly, children with a fourth or higher birth order had the highest complete vaccination rate (16%), thus suggesting that the birth order does not strongly predict vaccination completeness [22]. Maternal education and its impact on general health, including the vaccination status of children, is a known phenomenon [37,40]. Herein, our data reveal that children whose mothers had primary education had a lower rate of complete vaccination (6.45%) compared to those whose mothers had secondary (10%) or post-secondary education (10%) [26]. While the differences are not substantial, they indicate a potential trend towards higher education associated with better vaccination completeness. The importance of proper communication and education in improving health care outcomes cannot be ignored. In this study, we found that children whose caregivers did not receive an immunisation message in the last three months had a slightly lower rate of complete vaccination (12%) than those who received such messages (7%). This collaborates and highlights discrepancies in vaccination completeness among infants and children in comparison to different world regions [41,42,43]. In this case, it suggests that caregiver education and awareness campaigns may positively influence vaccination completeness [26].

Vaccine completeness over time is visually represented in Figure 1, showing the proportion of fully vaccinated, incompletely vaccinated, and unvaccinated children across various age groups. Noteworthy from the figure is that vaccination completeness tends to improve with age, with a notable increase in complete vaccination after the first few months of life. In fact, even though many associations between demographic and healthcare factors and vaccine completeness were not statistically significant, there were practical associations in reported trends. For example, the table results indicate a potential influence of employment status on childhood vaccination completeness. Children whose caregivers were employed had a higher rate of complete vaccination (12.65%) compared to those whose caregivers were unemployed (9.26%). This suggests a potential correlation between caregiver employment and the ability to adhere to the recommended vaccination schedule. The finding may be indicative of the role of financial stability and consistent access to healthcare services in ensuring timely and comprehensive Immunisation.

This highlights the importance of tracking and promoting timely vaccination during the early months and maintaining coverage as children age. Moreso, Figure 2 illustrates the distribution of children across different sub-districts in Cape Town, showcasing the number of records, fully vaccinated, and incompletely vaccinated participants. This figure highlights that the Tygerberg sub-district had the highest records, but many children were incompletely vaccinated. This suggests that specific sub-districts may require targeted interventions to improve vaccination completeness. Growing evidence is available to address these discrepancies, including suggestions for tailored interventions, including increasing vaccination facilities, community-based education, and targeted outreach efforts [44,45,46]. The study highlights the necessity to adapt public health policies and programs based on sub-district-specific challenges, directing resources to areas with high incompleteness rates. Ongoing monitoring and evaluation are crucial for assessing the effectiveness of interventions, ensuring timely and comprehensive Immunisation, and ultimately improving public health outcomes across Cape Town. Lastly, Figure 3 visually represents the knowledge about child vaccination among mothers or caregivers. It shows the distribution of caregivers with sufficient knowledge, insufficient knowledge, and those who did not answer questions about child vaccination. This affirms previous conversations [47,48] and emphasises the importance of assessing caregiver knowledge, which can impact vaccination decisions and adherence.

## 5. Conclusions

The data show positive trends in vaccination coverage for essential vaccines like BCG, OPV, RV, and DTaP-IPV-Hib-HepB in Cape Town. However, there is a drop in vaccination coverage as children grow older. Improvements are needed to maintain timely vaccinations, especially during the second year of life. Caregiver education and awareness campaigns are required to address knowledge gaps and misconceptions. These findings provide valuable guideposts for public health interventions in Cape Town to reduce the burden of vaccine-preventable diseases. While the sex of the child did not show significant differences in vaccination rates, it is essential to explore potential biological, cultural, or socio-economic reasons for the observed pattern where male children are more often incompletely vaccinated. The data from our study indicate that males were incompletely vaccinated compared to females, suggesting a slight disparity. Thus, investigating the underlying factors contributing to this difference, such as cultural beliefs, socio-economic conditions, or healthcare accessibility, could provide valuable insights for targeted interventions.

## Figures and Tables

**Table 1:** Vaccine dose taken and missed among children from birth to 23 months in Cape Town South Africa.

Eligible Age	Vaccine Offered (Dose)	Eligible	Vaccinated (%)	Not Vaccinated (%)
Birth	BCG	652	637 (97.70)	15 (2.30)
	OPV (0)	652	630 (96.63)	22 (3.37)
6 Weeks	OPV (1)	544	505 (92.83)	39 (7.17)
	RV (1)	544	537 (98.71)	7 (1.29)
	DTaP-IPV-Hib-HepB (1)	544	537 (98.71)	7 (1.29)
	PCV (1)	544	536 (98.53)	8 (1.47)
10 Weeks	DTaP-IPV-HIB-HepB (2)	475	468 (98.53)	7 (1.47)
14 Weeks	RV (2)	401	385 (96.00)	16 (3.99)
	DTaP-IPV-Hib-HepB (3)	401	392 (97.76)	9 (2.24)
	PCV (2)	401	389 (97.01)	12 (2.99)
6 Months	Measles (1)	341	325 (95.31)	16 (4.69)
9 Months	PCV (3)	246	238 (96.75)	9 (3.66)
12 Months	Measles (2)	169	153 (90.53)	16 (9.47)
18 Months	DTaP-IPV-HepB (4)	73	65 (89.04)	8 (10.69)

BCG, Bacille Calmette Guerin; DTaP-IPV-Hib-HepB, hexavalent vaccine (containing diphtheria, tetanus, pertussis, inactivated polio, Haemophilus influenzae type b, and hepatitis B vaccines); HPV, human papillomavirus vaccine; OPV, oral polio vaccine; PCV, pneumococcal conjugate vaccine; RV, rotavirus vaccine.

**Table 2:** Baseline characteristics associated with incomplete childhood vaccination among children from birth to 23 months attending primary healthcare facilities in Cape Town.

Variables	All (*n* = 652), *n* (%)	Fully (*n* = 66), *n* (%)	Incompletely (*n* = 586), *n* (%)
**CHILDREN CHARACTERISTICS**			
**Sex of child**			
Male	321, (49.23)	36, (11.21)	285, (88.79)
Female	331, (50.77)	30, (9.06)	301, (90.94)
**Age of child (months)**			
≤10	473, (75.55)	2, (0.42)	471, (99.58)
11–15	93, (14.26)	4, (4.30)	89, (95.70)
16–20	66, (10.12)	44, (66.67)	22, (33.33)
21–23	19, (2.91)	16, (84.21)	3, (15.79)
**Birth order**			
First	233, (35.74)	21, (9.01)	212, (89.83)
Second	228, (34.97)	18, (7.89)	210, (92.11)
Third	127, (19.48)	17, (13.39)	110, (86.61)
≥Fourth	64, (9.82)	10, (15.63)	54, (84.38)
**Birth weight**			
Average	413, (63.34)	44, (10.65)	369, (89.35)
Large	155, (23.77)	11, (7.10)	144, (92.90)
Small	80, (12.27)	10, (12.5)	70, (87.5)
**Reason for visit**			
Vaccination	449, (68.86)	41, (9.13)	408, (90.87)
Child treatment (sick)	113, (17.33)	16, (14.16)	97, (85.84)
GDGCU	64, (9.82)	4, (6.25)	60, (93.75)
Other	26, (3.99)	5, (19.23)	21, (80.77)
**RtHB asked by health worker**			
Yes	637, (97.70)	66, (10.36)	571, (89.64)
No	15, (2.30)		15, (100)
**Time of visit**			
Morning (8.01 a.m.–12 noon)	393, (60.28)	41, (10.43)	352, (89.57)
Afternoon (12.01–5:59 p.m.)	258, (39.57)	25, (9.69)	233, (90.31)
**MATERNAL OR/CAREGIVER CHARACTERISTICS**			
**Maternal age**			
16–24	181, (27.76)	14, (7.73)	167, (92.27)
25–34	321, (49.23)	35, (10.90)	286, (89.10)
35+	150, (23.01)	17, (11.33)	133, (88.67)
**Caregiver sex**			
Male	15, (2.30)	3, (20)	12, (80)
Female	630, (96.63)	63, (10)	567, (90)
**Marital Status**			
Married	221, (33.90)	25, (11.31)	196, (88.67)
Single	423, (64.88)	41, (9.69)	382, (90.31)
Divorced	4, (0.61)		4, (100)
Widowed	3, (0.46)		3, (100)
Other	1, (0.15)		1, (100)
**Relationship to child**			
Mother	604, (92.64)	57, (9.44)	547, (90.56)
Father	14, (2.15)	3, (21.43)	11, (78.57)
Sibling	34, (5.21)	6, (17.65)	28, (82.35)
**Maternal Education**			
NFE	2, (0.31)	1, (50.00)	1, (50.00)
Primary	31, (4.75)	2, (6.45)	29, (93.55)
Secondary	499, (68.87)	51, (10.22)	448, (8978)
Post-secondary	120, (18.40)	12, (10.00)	108, (90.10)
**Employment Status**			
Yes	166, (25.46)	21, (12.65)	145, (87.35)
No	486, (74.54)	45, (9.26)	441, 90.74)
**Maternal antenatal care**			
Yes	641, (98.31)	65, (10.02)	576, (88.75)
No	11, (1.69)	1, (9.09)	10, (90.91)
**Means of transport**			
Private	68, (10.43)	6, (8.82)	62, (91.18)
Public	205, (31.44)	22, (10.73)	183, (89.27)
Walk	379, (58.13)	38, (10.03)	341, (89.97)
**Immunisation message in last 3 months**			
Yes	402, (61.66)	48, (11.94)	354, (88.06)
No	249, (38.19)	18, (7.23)	231, (92.77)
**FACILITY CHARACTERISTICS**			
**Facility type**			
Clinic	326, (50)	36, (11.04)	290, (88.96)
CDC	324, (49.69)	30, (9.26)	294, (90.74)
**Facility Owner**			
Province	250, (38.34)	19, (7.60)	231, (92.40)
City	401, (61.50)	47, (11.72)	354, (88.28)
**Number of health workers**			
<20	148, (22.70)	19, (12.84)	129, (87.16)
20 < 50	271, (41.56)	27, (9.96)	244, (90.04)
≥50	230, (35.28)	20, (8.70)	210, (91.30)
**Vaccine stock-out in the past 3 months**			
Yes	141, (21.63)	14, (9.93)	127, (90.07)
No	509, (78.07)	52, (10.22)	455, (89.39)
**Vaccine cold-chain challenges in the past 3 months**			
Yes	39, (5.98)	4, (10.26)	35, (89.74)
No	612, (93.7)	62, (10.13)	550, (89.87)

CDC: Comprehensive; Fully: Fully vaccinated; Incompletely: Incompletely vaccinated; GDGCU: Growth development and general check-up; NFE: No Formal Education.

**Table 3:** Factors associated with vaccine completeness of children aged 0–23 months, five districts in Cape Town South Africa.

Variables	All (*n* = 652), *n* (%)	Measurement of Association			
		Bivariate Odds Ratio (95% CI)	*p*-Value	Adjusted Odds Ratio (95% CI)	*p*-Value
**CHILDREN CHARACTERISTICS**					
**Sex of child**					
Male	321, (49.23)	Ref		Ref	
Female	331, (50.77)	1.27 (0.76–2.11)	0.363	1.17 (0.47–2.91)	0.731
**Age of child (months)**					
≤10	473, (72.55)	Ref		Ref	
11–15	93, (14.26)	0.06 (0.01–0.31)	0.001	0.05 (0.009–0.31)	0.001
16–20	66, (10.12)	0.002 (0.0004–0.008)	0.000	0.001 (0.0003–0.0065)	0.000
21–23	19, (2.91)	0.0005 (0.0001–0.004)	0.000	0.0003 (0.00004–0.0029)	0.000
**Birth order**					
First	233, (335.74)	Ref		Ref	
Second	228, (34.97)	1.16 (0.60–2.23)	0.666	0.75 (0.25–2.28)	0.615
Third	127, (19.48)	0.64 (0.32–1.26)	0.200	0.68 (0.21–2.23)	0.522
≥Fourth	64, (9.82)	0.53 (0.24–1.20)	0.130	0.33 (0.07–1.56)	0.162
**Birth Weight**					
Average	413, (63.34)	Ref		Ref	
Large	155, (23.77)	1.56 (0.78–3.11)	0.205	1.83 (0.60–5.56)	0.288
Small	80, (12.27)	0.82 (0.40–1.71)	0.602	0.75 (0.20–2.82)	0.671
Not specified	4, (0.61)	0.48 (0.52–4.36)	0.512	1.35 (0.014–127.99)	0.897
**Reason for visit**					
Vaccination	449, (68.87)	Ref		Ref	
Non-vaccination	203, (31.13)	0.71 (0.42–1.21)	0.214	1.86 (0.68–5.12)	0.229
**Time of visit**					
Morning (8.01 a.m.–12 noon)	393, (60.28)	Ref		Ref	
Afternoon	258, (39.57)	1.09 (0.64–1.83)	0.759	1.69 (0.66–4.30)	0.272
**MATERNAL OR/CAREGIVER CHARACTERISTICS**					
**Maternal age**					
16–24	181, (27.76)	Ref		Ref	
25–34	321, (49.23)	0.69 (0.39–1.31)	0.253	0.76 (0.39–1.48)	0.424
35+	150, (23.01)	0.66 (0.31–1.39)	0.266	0.83 (0.37–1.86)	0.646
**Caregiver sex**					
Male	15, (2.30)	Ref		Ref	
Female	630, (96.63)	2.25 (0.61–8.19)	0.219	0.76 (0.015–37.25)	0.889
Not specified	7, (1.07)	empty			
**Marital Status**					
Married	221, (33.90)	Ref		Ref	
Single	423, (64.88)	1.19 (0.70–2.01)	0.520	1.07 (0.61–1.89)	0.803
Divorced	4, (0.61)	empty			
Widowed	3, (0.46)	empty			
Other	1, (0.15)	empty			
**Relationship to child**					
Mother	604, (92.64)	Ref		Ref	
Father	14, (2.15)	0.38 (0.10–1.41)	0.149	0.35 (0.01–17.74)	0.602
Sibling	34, (5.21)	0.49 (0.19–1.22)	0.126	0.38 (0.14–1.09)	0.073
**Maternal education**					
No Formal Education	2, (0.31)	Ref		Ref	
Primary	31, (4.75)	8.78 (0.54–142.58)	0.126	6.12 (0.36–105.26)	0.212
Secondary	499, (76.53)	14.5 (0.64–328.46)	0.093	10.28 (0.44–241.42)	0.148
Post-secondary	120, (18.40)	9 (0.53–153.31)	0.129	6.57 (0.36–120.32)	0.205
**Employment status**					
Yes	166, (25.46)	Ref		Ref	
No	486, (74.54)	1.42 (0.82–2.46)	0.213	1.31 (0.72–2.38)	0.379
**Maternal antenatal care**					
Yes	649, (99.54)	Ref		Ref	
No	11, (1.69)	0.79 (0.096–6.52)	0.827	1.0 (0.11–9.51)	1.0
**Means of transport**					
Private	68, (10.43)	Ref		Ref	
Public	205, (31.44)	0.80 (0.31–2.08)	0.654	0.73 (0.28–1.92)	0.521
Walk	379, (58.13)	0.87 (0.35–2.14)	0.759	0.71 (0.27–1.83)	0.478
**Immunisation message in last 3 months**					
Yes	402, (61.66)	Ref		Ref	
No	250, (38.34)	1.74 (0.99–3.07)	0.055	1.77 (0.98–3.20)	0.058
**FACILITY CHARACTERISTICS**					
**Facility type**					
Clinic	326, (50)	Ref		Ref	
CDC	324, (49.69)	1.22 (0.73–2.03)	0.452	0.76 (0.35–1.65)	0.481
Not specified	2, (0.31)	Empty		Empty	
**Facility Owners**					
Province	250, (38.34)	Ref		Ref	
City	401, (61.50)	0.62 (0.35–1.08)	0.093	0.53 (0.21–1.34)	0.179
**Number of health workers**					
<20	148, (22.70)	Ref		Ref	
20 < 50	271, (41.56)	1.33 (0.71–2.49)	0.369	1.10 (0.54–2.26)	0.794
≥50	230, (35.28)	1.55 (0.80–3.00)	0.199	1.27 (0.46–3.79)	0.669
Not specified	3, (0.46)	Empty		Empty	
**Vaccine stock-out in the past 3 months**					
Yes	141, (21.63)	Ref		Ref	
No	509, (78.07)	0.97 (0.52–1.80)	0.920	1.13 (0.41–3.09)	0.811
Not specified	2, (0.31)	empty		Empty	
**Vaccine cold-chain challenges in the past 3 months**					
Yes	39, (5.98)	Ref		Ref	
No	612, (93.87)	0.98 (0.34–2.86)	0.976	0.89 (0.27–2.86)	0.841
Not specified	1, (0.15)	empty		Empty	
**Immunisation waiting time**					
Less than 30 min	165, (25.31)	Ref		Ref	
>30	327, (50.15)	0.94 (0.51–1.75)	0.854	0.93 (0.49–1.76)	0.829
Not specified	115, (17.64)	0.92 (0.42–2.03)	0.840	0.81 (0.35–1.84)	0.614

CDC: Comprehensive.

## Data Availability

All data used in this study are included as part of the manuscript. Raw data can be requested from the corresponding author.
